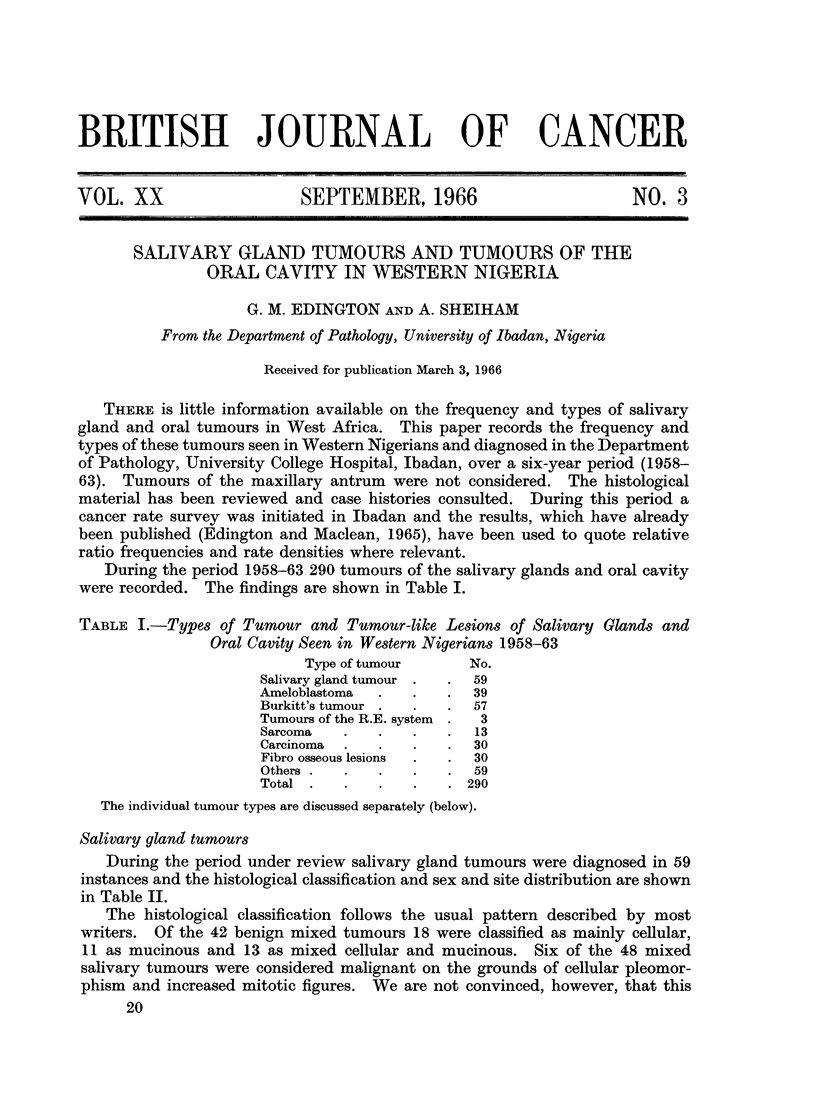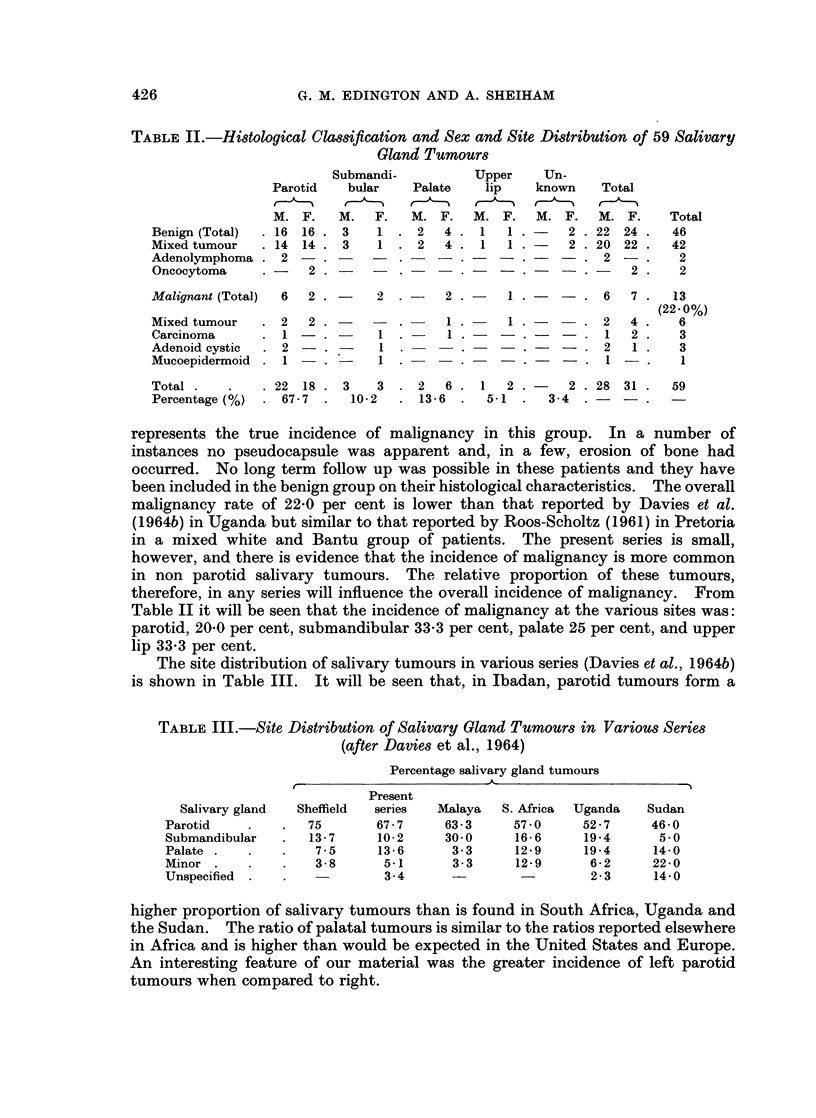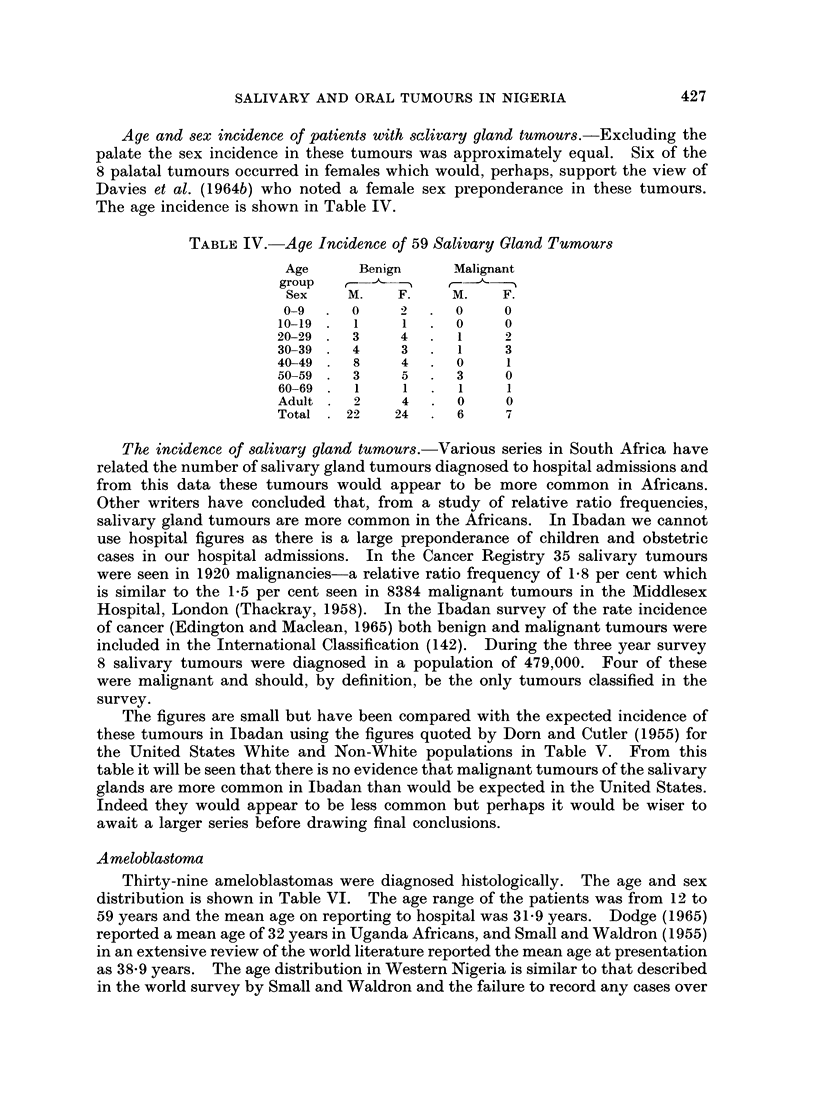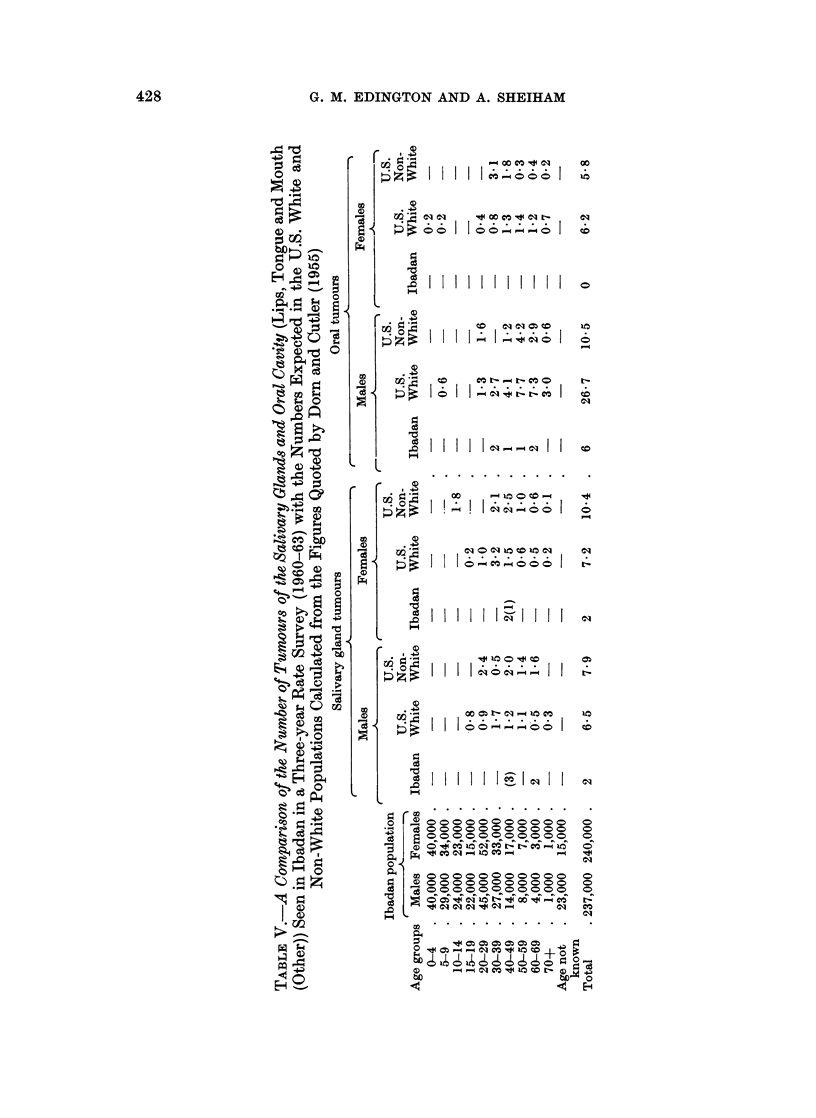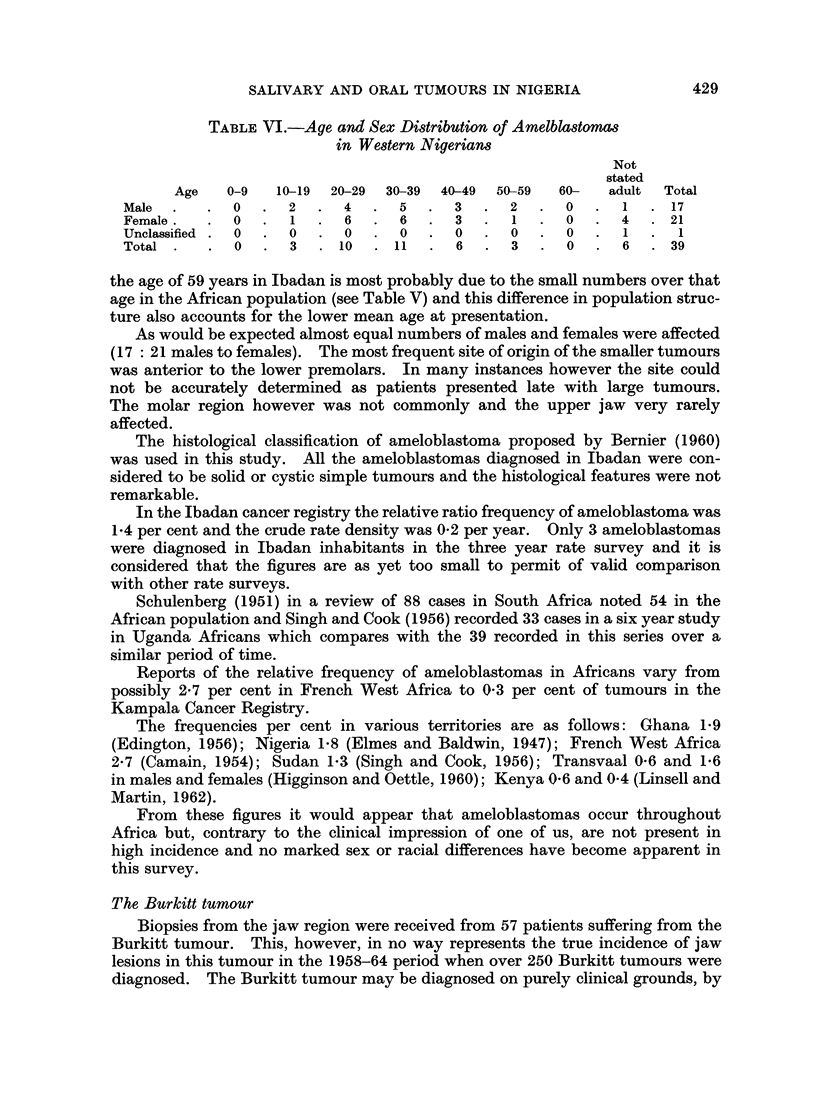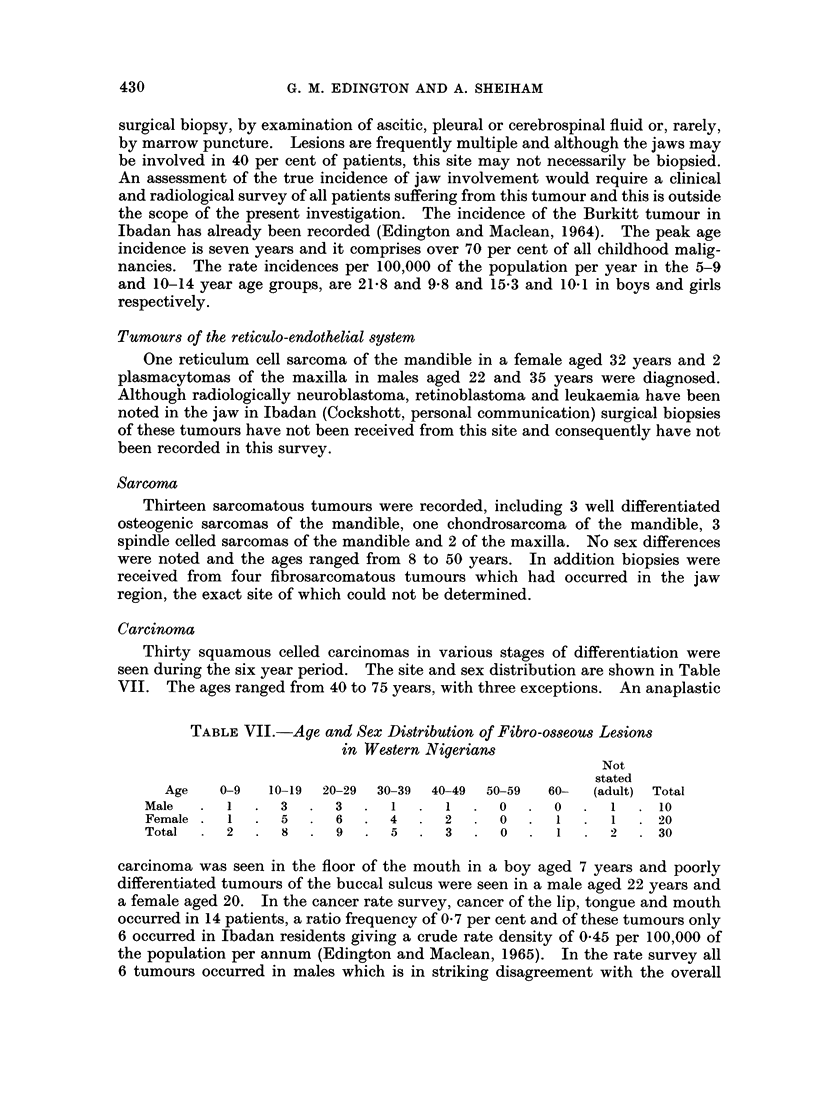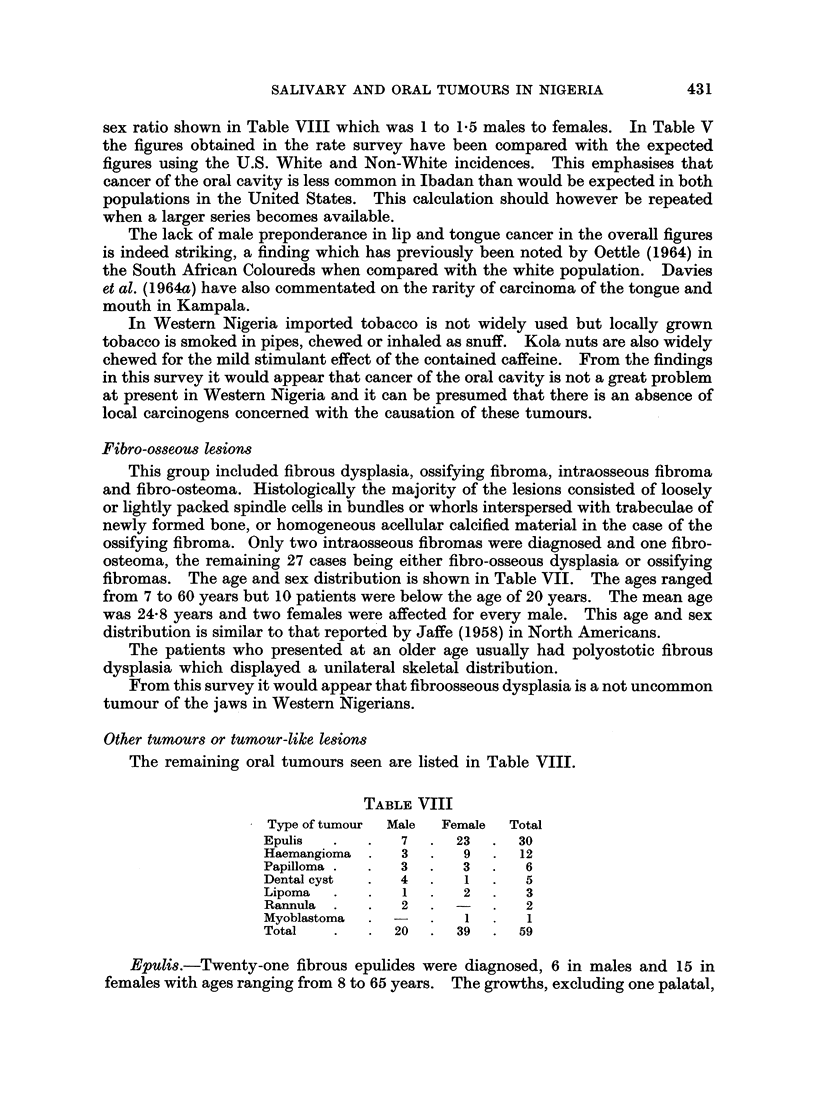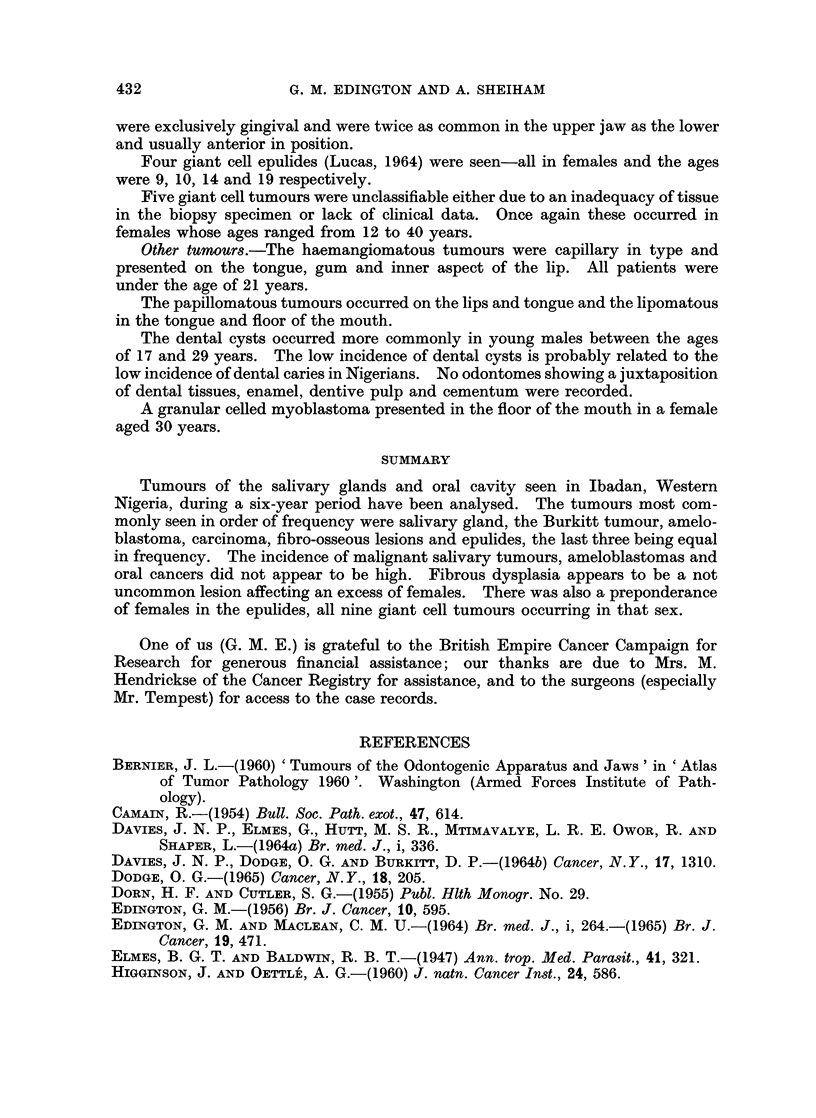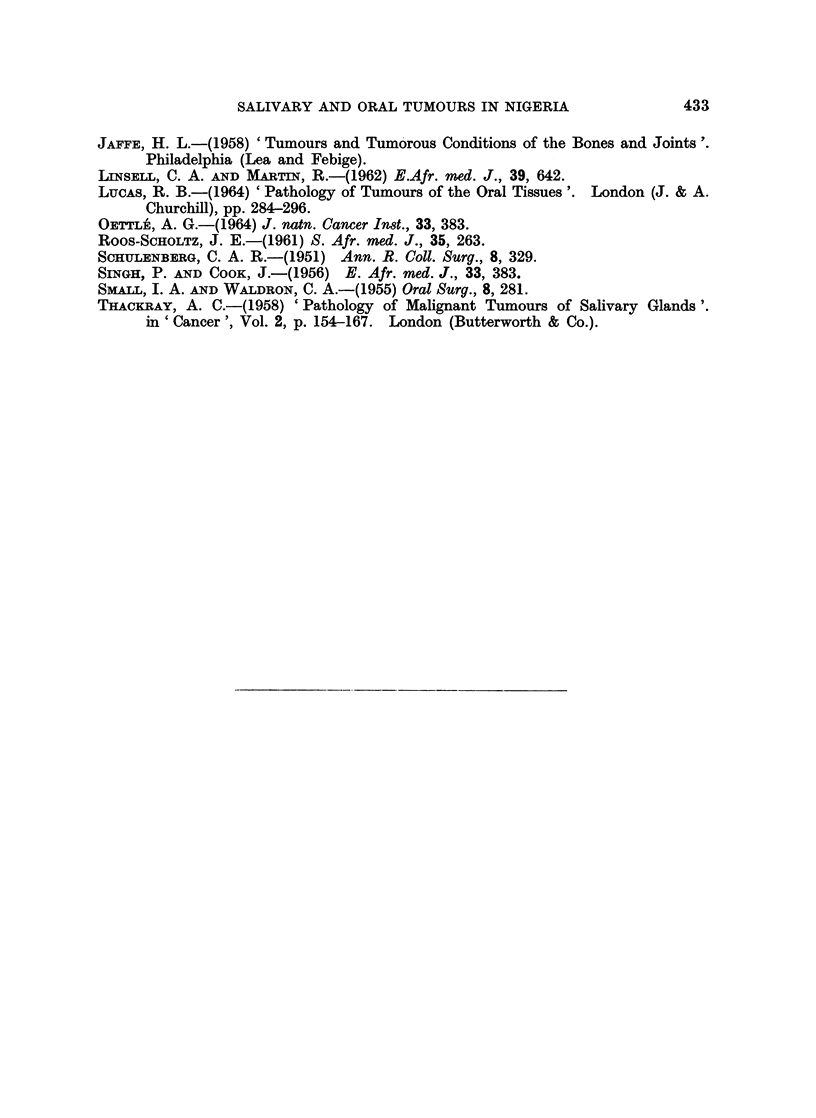# Salivary gland tumours and tumours of the oral cavity in western Nigeria.

**DOI:** 10.1038/bjc.1966.51

**Published:** 1966-09

**Authors:** G. M. Edington, A. Sheiham


### BRITISH JOURNAL OF CANCER

VOL. XX SEPTEMBER, 1966 NO. 3

### SALIVARY GLAND TUMOURS AND TUMOURS OF THE ORAL CAVITY IN WESTERN NIGERIA

G. M. EDINGTON AND A. SHEIHAM

*From the Department of Pathology, University of Ibadan, Nigeria*

Received for publication March 3, 1966

THERE is little information available on the frequency and types of salivary gland and oral tumours in West Africa. This paper records the frequency and types of these tumours seen in Western Nigerians and diagnosed in the Department of Pathology, University College Hospital, Ibadan, over a six-year period (1958–63). Tumours of the maxillary antrum were not considered. The histological material has been reviewed and case histories consulted. During this period a cancer rate survey was initiated in Ibadan and the results, which have already been published (Edington and Maclean, 1965), have been used to quote relative ratio frequencies and rate densities where relevant.

During the period 1958–63 290 tumours of the salivary glands and oral cavity were recorded. The findings are shown in Table I.

TABLE I.—*Types of Tumour and Tumour-like Lesions of Salivary Glands and Oral Cavity Seen in Western Nigerians* 1958–63

| Type of tumour | No. |
|---|---|
| Salivary gland tumour | 59 |
| Ameloblastoma | 39 |
| Burkitt's tumour | 57 |
| Tumours of the R.E. system | 3 |
| Sarcoma | 13 |
| Carcinoma | 30 |
| Fibro osseous lesions | 30 |
| Others | 59 |
| Total | 290 |

The individual tumour types are discussed separately (below).

### *Salivary gland tumours*

During the period under review salivary gland tumours were diagnosed in 59 instances and the histological classification and sex and site distribution are shown in Table II.

The histological classification follows the usual pattern described by most writers. Of the 42 benign mixed tumours 18 were classified as mainly cellular, 11 as mucinous and 13 as mixed cellular and mucinous. Six of the 48 mixed salivary tumours were considered malignant on the grounds of cellular pleomorphism and increased mitotic figures. We are not convinced, however, that this

TABLE II.—*Histological Classification and Sex and Site Distribution of* 59 *Salivary Gland Tumours*

| | Parotid | | Submandibular | | Palate | | Upper lip | | Unknown | | Total | | |
|---|---|---|---|---|---|---|---|---|---|---|---|---|---|
| | M. | F. | M. | F. | M. | F. | M. | F. | M. | F. | M. | F. | Total |
| Benign (Total) | 16 | 16 | 3 | 1 | 2 | 4 | 1 | 1 | — | 2 | 22 | 24 | 46 |
| Mixed tumour | 14 | 14 | 3 | 1 | 2 | 4 | 1 | 1 | — | 2 | 20 | 22 | 42 |
| Adenolymphoma | 2 | — | — | — | — | — | — | — | — | — | 2 | — | 2 |
| Oncocytoma | — | 2 | — | — | — | — | — | — | — | — | — | 2 | 2 |
| *Malignant* (Total) | 6 | 2 | — | 2 | — | 2 | — | 1 | — | — | 6 | 7 | 13 (22·0%) |
| Mixed tumour | 2 | 2 | — | — | — | 1 | — | 1 | — | — | 2 | 4 | 6 |
| Carcinoma | 1 | — | — | 1 | — | 1 | — | — | — | — | 1 | 2 | 3 |
| Adenoid cystic | 2 | — | — | 1 | — | — | — | — | — | — | 2 | 1 | 3 |
| Mucoepidermoid | 1 | — | — | 1 | — | — | — | — | — | — | 1 | — | 1 |
| Total | 22 | 18 | 3 | 3 | 2 | 6 | 1 | 2 | — | 2 | 28 | 31 | 59 |
| Percentage (%) | 67·7 | | 10·2 | | 13·6 | | 5·1 | | 3·4 | | — | — | — |

represents the true incidence of malignancy in this group. In a number of instances no pseudocapsule was apparent and, in a few, erosion of bone had occurred. No long term follow up was possible in these patients and they have been included in the benign group on their histological characteristics. The overall malignancy rate of 22·0 per cent is lower than that reported by Davies *et al.* (1964*b*) in Uganda but similar to that reported by Roos-Scholtz (1961) in Pretoria in a mixed white and Bantu group of patients. The present series is small, however, and there is evidence that the incidence of malignancy is more common in non parotid salivary tumours. The relative proportion of these tumours, therefore, in any series will influence the overall incidence of malignancy. From Table II it will be seen that the incidence of malignancy at the various sites was: parotid, 20·0 per cent, submandibular 33·3 per cent, palate 25 per cent, and upper lip 33·3 per cent.

The site distribution of salivary tumours in various series (Davies *et al.*, 1964*b*) is shown in Table III. It will be seen that, in Ibadan, parotid tumours form a

TABLE III.—*Site Distribution of Salivary Gland Tumours in Various Series* (*after Davies* et al., 1964)

| | Percentage salivary gland tumours | | | | | |
|---|---|---|---|---|---|---|
| Salivary gland | Sheffield | Present series | Malaya | S. Africa | Uganda | Sudan |
| Parotid | 75 | 67·7 | 63·3 | 57·0 | 52·7 | 46·0 |
| Submandibular | 13·7 | 10·2 | 30·0 | 16·6 | 19·4 | 5·0 |
| Palate | 7·5 | 13·6 | 3·3 | 12·9 | 19·4 | 14·0 |
| Minor | 3·8 | 5·1 | 3·3 | 12·9 | 6·2 | 22·0 |
| Unspecified | — | 3·4 | — | — | 2·3 | 14·0 |

higher proportion of salivary tumours than is found in South Africa, Uganda and the Sudan. The ratio of palatal tumours is similar to the ratios reported elsewhere in Africa and is higher than would be expected in the United States and Europe. An interesting feature of our material was the greater incidence of left parotid tumours when compared to right.

*Age and sex incidence of patients with sclivary gland tumours.*—Excluding the palate the sex incidence in these tumours was approximately equal. Six of the 8 palatal tumours occurred in females which would, perhaps, support the view of Davies *et al.* (1964*b*) who noted a female sex preponderance in these tumours. The age incidence is shown in Table IV.

TABLE IV.—*Age Incidence of* 59 *Salivary Gland Tumours*

| Age group | Benign | | Malignant | |
|---|---|---|---|---|
| Sex | M. | F. | M. | F. |
| 0–9 | 0 | 2 | 0 | 0 |
| 10–19 | 1 | 1 | 0 | 0 |
| 20–29 | 3 | 4 | 1 | 2 |
| 30–39 | 4 | 3 | 1 | 3 |
| 40–49 | 8 | 4 | 0 | 1 |
| 50–59 | 3 | 5 | 3 | 0 |
| 60–69 | 1 | 1 | 1 | 1 |
| Adult | 2 | 4 | 0 | 0 |
| Total | 22 | 24 | 6 | 7 |

*The incidence of salivary gland tumours.*—Various series in South Africa have related the number of salivary gland tumours diagnosed to hospital admissions and from this data these tumours would appear to be more common in Africans. Other writers have concluded that, from a study of relative ratio frequencies, salivary gland tumours are more common in the Africans. In Ibadan we cannot use hospital figures as there is a large preponderance of children and obstetric cases in our hospital admissions. In the Cancer Registry 35 salivary tumours were seen in 1920 malignancies—a relative ratio frequency of 1·8 per cent which is similar to the 1·5 per cent seen in 8384 malignant tumours in the Middlesex Hospital, London (Thackray, 1958). In the Ibadan survey of the rate incidence of cancer (Edington and Maclean, 1965) both benign and malignant tumours were included in the International Classification (142). During the three year survey 8 salivary tumours were diagnosed in a population of 479,000. Four of these were malignant and should, by definition, be the only tumours classified in the survey.

The figures are small but have been compared with the expected incidence of these tumours in Ibadan using the figures quoted by Dorn and Cutler (1955) for the United States White and Non-White populations in Table V. From this table it will be seen that there is no evidence that malignant tumours of the salivary glands are more common in Ibadan than would be expected in the United States. Indeed they would appear to be less common but perhaps it would be wiser to await a larger series before drawing final conclusions.

### *Ameloblastoma*

Thirty-nine ameloblastomas were diagnosed histologically. The age and sex distribution is shown in Table VI. The age range of the patients was from 12 to 59 years and the mean age on reporting to hospital was 31·9 years. Dodge (1965) reported a mean age of 32 years in Uganda Africans, and Small and Waldron (1955) in an extensive review of the world literature reported the mean age at presentation as 38·9 years. The age distribution in Western Nigeria is similar to that described in the world survey by Small and Waldron and the failure to record any cases over

TABLE V.—*A Comparison of the Number of Tumours of the Salivary Glands and Oral Cavity* (Lips, Tongue and Mouth (Other)) Seen in Ibadan in a Three-year Rate Survey (1960–63) with the Numbers Expected in the U.S. White and Non-White Populations Calculated from the Figures Quoted by Dorn and Cutler (1955)

| | Ibadan population | | Salivary gland tumours | | | | | | Oral tumours | | | | | |
|---|---|---|---|---|---|---|---|---|---|---|---|---|---|---|
| | | | Males | | | Females | | | Males | | | Females | | |
| Age groups | Males | Females | Ibadan | U.S. White | U.S. Non-White | Ibadan | U.S. White | U.S. Non-White | Ibadan | U.S. White | U.S. Non-White | Ibadan | U.S. White | U.S. Non-White |
| 0–4 | 40,000 | 40,000 | — | — | — | — | — | — | — | — | — | — | 0·2 | — |
| 5–9 | 29,000 | 34,000 | — | — | — | — | — | — | — | 0·6 | — | — | 0·2 | — |
| 10–14 | 24,000 | 23,000 | — | — | — | — | — | 1·8 | — | — | — | — | — | — |
| 15–19 | 22,000 | 15,000 | — | 0·8 | — | — | 0·2 | — | — | — | — | — | — | — |
| 20–29 | 45,000 | 52,000 | — | 0·9 | 2·4 | — | 1·0 | — | — | 1·3 | 1·6 | — | 0·4 | — |
| 30–39 | 27,000 | 33,000 | — | 1·7 | 0·5 | — | 3·2 | 2·1 | 2 | 2·7 | — | — | 0·8 | 3·1 |
| 40–49 | 14,000 | 17,000 | (3) | 1·2 | 2·0 | 2(1) | 1·5 | 2·5 | 1 | 4·1 | 1·2 | — | 1·3 | 1·8 |
| 50–59 | 8,000 | 7,000 | — | 1·1 | 1·4 | — | 0·6 | 1·0 | 1 | 7·7 | 4·2 | — | 1·4 | 0·3 |
| 60–69 | 4,000 | 3,000 | 2 | 0·5 | 1·6 | — | 0·5 | 0·6 | 2 | 7·3 | 2·9 | — | 1·2 | 0·4 |
| 70+ | 1,000 | 1,000 | — | 0·3 | — | — | 0·2 | 0·1 | — | 3·0 | 0·6 | — | 0·7 | 0·2 |
| Age not known | 23,000 | 15,000 | — | — | — | — | — | — | — | — | — | — | — | — |
| Total | 237,000 | 240,000 | 2 | 6·5 | 7·9 | 2 | 7·2 | 10·4 | 6 | 26·7 | 10·5 | 0 | 6·2 | 5·8 |

TABLE VI.—*Age and Sex Distribution of Amelblastomas in Western Nigerians*

| Age | 0–9 | 10–19 | 20–29 | 30–39 | 40–49 | 50–59 | 60– | Not stated adult | Total |
|---|---|---|---|---|---|---|---|---|---|
| Male | 0 | 2 | 4 | 5 | 3 | 2 | 0 | 1 | 17 |
| Female | 0 | 1 | 6 | 6 | 3 | 1 | 0 | 4 | 21 |
| Unclassified | 0 | 0 | 0 | 0 | 0 | 0 | 0 | 1 | 1 |
| Total | 0 | 3 | 10 | 11 | 6 | 3 | 0 | 6 | 39 |

the age of 59 years in Ibadan is most probably due to the small numbers over that age in the African population (see Table V) and this difference in population structure also accounts for the lower mean age at presentation.

As would be expected almost equal numbers of males and females were affected (17 : 21 males to females). The most frequent site of origin of the smaller tumours was anterior to the lower premolars. In many instances however the site could not be accurately determined as patients presented late with large tumours. The molar region however was not commonly and the upper jaw very rarely affected.

The histological classification of ameloblastoma proposed by Bernier (1960) was used in this study. All the ameloblastomas diagnosed in Ibadan were considered to be solid or cystic simple tumours and the histological features were not remarkable.

In the Ibadan cancer registry the relative ratio frequency of ameloblastoma was 1·4 per cent and the crude rate density was 0·2 per year. Only 3 ameloblastomas were diagnosed in Ibadan inhabitants in the three year rate survey and it is considered that the figures are as yet too small to permit of valid comparison with other rate surveys.

Schulenberg (1951) in a review of 88 cases in South Africa noted 54 in the African population and Singh and Cook (1956) recorded 33 cases in a six year study in Uganda Africans which compares with the 39 recorded in this series over a similar period of time.

Reports of the relative frequency of ameloblastomas in Africans vary from possibly 2·7 per cent in French West Africa to 0·3 per cent of tumours in the Kampala Cancer Registry.

The frequencies per cent in various territories are as follows: Ghana 1·9 (Edington, 1956); Nigeria 1·8 (Elmes and Baldwin, 1947); French West Africa 2·7 (Camain, 1954); Sudan 1·3 (Singh and Cook, 1956); Transvaal 0·6 and 1·6 in males and females (Higginson and Oettle, 1960); Kenya 0·6 and 0·4 (Linsell and Martin, 1962).

From these figures it would appear that ameloblastomas occur throughout Africa but, contrary to the clinical impression of one of us, are not present in high incidence and no marked sex or racial differences have become apparent in this survey.

*The Burkitt tumour*

Biopsies from the jaw region were received from 57 patients suffering from the Burkitt tumour. This, however, in no way represents the true incidence of jaw lesions in this tumour in the 1958–64 period when over 250 Burkitt tumours were diagnosed. The Burkitt tumour may be diagnosed on purely clinical grounds, by surgical biopsy, by examination of ascitic, pleural or cerebrospinal fluid or, rarely, by marrow puncture. Lesions are frequently multiple and although the jaws may be involved in 40 per cent of patients, this site may not necessarily be biopsied. An assessment of the true incidence of jaw involvement would require a clinical and radiological survey of all patients suffering from this tumour and this is outside the scope of the present investigation. The incidence of the Burkitt tumour in Ibadan has already been recorded (Edington and Maclean, 1964). The peak age incidence is seven years and it comprises over 70 per cent of all childhood malignancies. The rate incidences per 100,000 of the population per year in the 5–9 and 10–14 year age groups, are 21·8 and 9·8 and 15·3 and 10·1 in boys and girls respectively.

*Tumours of the reticulo-endothelial system*

One reticulum cell sarcoma of the mandible in a female aged 32 years and 2 plasmacytomas of the maxilla in males aged 22 and 35 years were diagnosed. Although radiologically neuroblastoma, retinoblastoma and leukaemia have been noted in the jaw in Ibadan (Cockshott, personal communication) surgical biopsies of these tumours have not been received from this site and consequently have not been recorded in this survey.

*Sarcoma*

Thirteen sarcomatous tumours were recorded, including 3 well differentiated osteogenic sarcomas of the mandible, one chondrosarcoma of the mandible, 3 spindle celled sarcomas of the mandible and 2 of the maxilla. No sex differences were noted and the ages ranged from 8 to 50 years. In addition biopsies were received from four fibrosarcomatous tumours which had occurred in the jaw region, the exact site of which could not be determined.

*Carcinoma*

Thirty squamous celled carcinomas in various stages of differentiation were seen during the six year period. The site and sex distribution are shown in Table VII. The ages ranged from 40 to 75 years, with three exceptions. An anaplastic

TABLE VII.—*Age and Sex Distribution of Fibro-osseous Lesions in Western Nigerians*

| Age | 0–9 | 10–19 | 20–29 | 30–39 | 40–49 | 50–59 | 60– | Not stated (adult) | Total |
|---|---|---|---|---|---|---|---|---|---|
| Male | 1 | 3 | 3 | 1 | 1 | 0 | 0 | 1 | 10 |
| Female | 1 | 5 | 6 | 4 | 2 | 0 | 1 | 1 | 20 |
| Total | 2 | 8 | 9 | 5 | 3 | 0 | 1 | 2 | 30 |

carcinoma was seen in the floor of the mouth in a boy aged 7 years and poorly differentiated tumours of the buccal sulcus were seen in a male aged 22 years and a female aged 20. In the cancer rate survey, cancer of the lip, tongue and mouth occurred in 14 patients, a ratio frequency of 0·7 per cent and of these tumours only 6 occurred in Ibadan residents giving a crude rate density of 0·45 per 100,000 of the population per annum (Edington and Maclean, 1965). In the rate survey all 6 tumours occurred in males which is in striking disagreement with the overall sex ratio shown in Table VIII which was 1 to 1·5 males to females. In Table V the figures obtained in the rate survey have been compared with the expected figures using the U.S. White and Non-White incidences. This emphasises that cancer of the oral cavity is less common in Ibadan than would be expected in both populations in the United States. This calculation should however be repeated when a larger series becomes available.

The lack of male preponderance in lip and tongue cancer in the overall figures is indeed striking, a finding which has previously been noted by Oettle (1964) in the South African Coloureds when compared with the white population. Davies *et al.* (1964*a*) have also commentated on the rarity of carcinoma of the tongue and mouth in Kampala.

In Western Nigeria imported tobacco is not widely used but locally grown tobacco is smoked in pipes, chewed or inhaled as snuff. Kola nuts are also widely chewed for the mild stimulant effect of the contained caffeine. From the findings in this survey it would appear that cancer of the oral cavity is not a great problem at present in Western Nigeria and it can be presumed that there is an absence of local carcinogens concerned with the causation of these tumours.

*Fibro-osseous lesions*

This group included fibrous dysplasia, ossifying fibroma, intraosseous fibroma and fibro-osteoma. Histologically the majority of the lesions consisted of loosely or lightly packed spindle cells in bundles or whorls interspersed with trabeculae of newly formed bone, or homogeneous acellular calcified material in the case of the ossifying fibroma. Only two intraosseous fibromas were diagnosed and one fibro-osteoma, the remaining 27 cases being either fibro-osseous dysplasia or ossifying fibromas. The age and sex distribution is shown in Table VII. The ages ranged from 7 to 60 years but 10 patients were below the age of 20 years. The mean age was 24·8 years and two females were affected for every male. This age and sex distribution is similar to that reported by Jaffe (1958) in North Americans.

The patients who presented at an older age usually had polyostotic fibrous dysplasia which displayed a unilateral skeletal distribution.

From this survey it would appear that fibroosseous dysplasia is a not uncommon tumour of the jaws in Western Nigerians.

*Other tumours or tumour-like lesions*

The remaining oral tumours seen are listed in Table VIII.

TABLE VIII

| Type of tumour | Male | Female | Total |
|---|---|---|---|
| Epulis | 7 | 23 | 30 |
| Haemangioma | 3 | 9 | 12 |
| Papilloma | 3 | 3 | 6 |
| Dental cyst | 4 | 1 | 5 |
| Lipoma | 1 | 2 | 3 |
| Rannula | 2 | — | 2 |
| Myoblastoma | — | 1 | 1 |
| Total | 20 | 39 | 59 |

*Epulis*.—Twenty-one fibrous epulides were diagnosed, 6 in males and 15 in females with ages ranging from 8 to 65 years. The growths, excluding one palatal, were exclusively gingival and were twice as common in the upper jaw as the lower and usually anterior in position.

Four giant cell epulides (Lucas, 1964) were seen—all in females and the ages were 9, 10, 14 and 19 respectively.

Five giant cell tumours were unclassifiable either due to an inadequacy of tissue in the biopsy specimen or lack of clinical data. Once again these occurred in females whose ages ranged from 12 to 40 years.

*Other tumours*.—The haemangiomatous tumours were capillary in type and presented on the tongue, gum and inner aspect of the lip. All patients were under the age of 21 years.

The papillomatous tumours occurred on the lips and tongue and the lipomatous in the tongue and floor of the mouth.

The dental cysts occurred more commonly in young males between the ages of 17 and 29 years. The low incidence of dental cysts is probably related to the low incidence of dental caries in Nigerians. No odontomes showing a juxtaposition of dental tissues, enamel, dentive pulp and cementum were recorded.

A granular celled myoblastoma presented in the floor of the mouth in a female aged 30 years.

### SUMMARY

Tumours of the salivary glands and oral cavity seen in Ibadan, Western Nigeria, during a six-year period have been analysed. The tumours most commonly seen in order of frequency were salivary gland, the Burkitt tumour, ameloblastoma, carcinoma, fibro-osseous lesions and epulides, the last three being equal in frequency. The incidence of malignant salivary tumours, ameloblastomas and oral cancers did not appear to be high. Fibrous dysplasia appears to be a not uncommon lesion affecting an excess of females. There was also a preponderance of females in the epulides, all nine giant cell tumours occurring in that sex.

One of us (G. M. E.) is grateful to the British Empire Cancer Campaign for Research for generous financial assistance; our thanks are due to Mrs. M. Hendrickse of the Cancer Registry for assistance, and to the surgeons (especially Mr. Tempest) for access to the case records.

### REFERENCES

BERNIER, J. L.—(1960) ‘ Tumours of the Odontogenic Apparatus and Jaws ’ in ‘ Atlas of Tumor Pathology 1960 ’. Washington (Armed Forces Institute of Pathology).

CAMAIN, R.—(1954) *Bull. Soc. Path. exot.*, **47**, 614.

DAVIES, J. N. P., ELMES, G., HUTT, M. S. R., MTIMAVALYE, L. R. E. OWOR, R. AND SHAPER, L.—(1964*a*) *Br. med. J.*, i, 336.

DAVIES, J. N. P., DODGE, O. G. AND BURKITT, D. P.—(1964*b*) *Cancer, N.Y.*, **17**, 1310.

DODGE, O. G.—(1965) *Cancer, N.Y.*, **18**, 205.

DORN, H. F. AND CUTLER, S. G.—(1955) *Publ. Hlth Monogr.* No. 29.

EDINGTON, G. M.—(1956) *Br. J. Cancer*, **10**, 595.

EDINGTON, G. M. AND MACLEAN, C. M. U.—(1964) *Br. med. J.*, i, 264.—(1965) *Br. J. Cancer*, **19**, 471.

ELMES, B. G. T. AND BALDWIN, R. B. T.—(1947) *Ann. trop. Med. Parasit.*, **41**, 321.

HIGGINSON, J. AND OETTLÉ, A. G.—(1960) *J. natn. Cancer Inst.*, **24**, 586.

JAFFE, H. L.—(1958) 'Tumours and Tumorous Conditions of the Bones and Joints'. Philadelphia (Lea and Febige).
LINSELL, C. A. AND MARTIN, R.—(1962) *E.Afr. med. J.*, **39**, 642.
LUCAS, R. B.—(1964) 'Pathology of Tumours of the Oral Tissues'. London (J. & A. Churchill), pp. 284–296.
OETTLÉ, A. G.—(1964) *J. natn. Cancer Inst.*, **33**, 383.
ROOS-SCHOLTZ, J. E.—(1961) *S. Afr. med. J.*, **35**, 263.
SCHULENBERG, C. A. R.—(1951) *Ann. R. Coll. Surg.*, **8**, 329.
SINGH, P. AND COOK, J.—(1956) *E. Afr. med. J.*, **33**, 383.
SMALL, I. A. AND WALDRON, C. A.—(1955) *Oral Surg.*, **8**, 281.
THACKRAY, A. C.—(1958) 'Pathology of Malignant Tumours of Salivary Glands'. in 'Cancer', Vol. **2**, p. 154–167. London (Butterworth & Co.).